# Health Information National Trends Survey in American Sign Language (HINTS-ASL): Protocol for the Cultural Adaptation and Linguistic Validation of a National Survey

**DOI:** 10.2196/resprot.8067

**Published:** 2017-09-13

**Authors:** Poorna Kushalnagar, Raychelle Harris, Raylene Paludneviciene, TraciAnn Hoglind

**Affiliations:** ^1^ Deaf Health Communication and Quality of Life Center Department of Psychology Gallaudet University Washington, DC United States; ^2^ Department of American Sign Language and Deaf Studies Gallaudet University Washington, DC United States; ^3^ Department of Psychology Gallaudet University Washington, DC United States; ^4^ Deaf Health Communication and Quality of Life Center Gallaudet University Washington, DC United States

**Keywords:** HINTS, health information seeking, American Sign Language, signed language, cultural adaptation, translation, health surveillance, survey

## Abstract

**Background:**

The Health Information National Trends Survey (HINTS) collects nationally representative data about the American’s public use of health-related information. This survey is available in English and Spanish, but not in American Sign Language (ASL). Thus, the exclusion of ASL users from these national health information survey studies has led to a significant gap in knowledge of Internet usage for health information access in this underserved and understudied population.

**Objective:**

The objectives of this study are (1) to culturally adapt and linguistically translate the HINTS items to ASL (HINTS-ASL); and (2) to gather information about deaf people’s health information seeking behaviors across technology-mediated platforms.

**Methods:**

We modified the standard procedures developed at the US National Center for Health Statistics Cognitive Survey Laboratory to culturally adapt and translate HINTS items to ASL. Cognitive interviews were conducted to assess clarity and delivery of these HINTS-ASL items. Final ASL video items were uploaded to a protected online survey website. The HINTS-ASL online survey has been administered to over 1350 deaf adults (ages 18 to 90 and up) who use ASL. Data collection is ongoing and includes deaf adult signers across the United States.

**Results:**

Some items from HINTS item bank required cultural adaptation for use with deaf people who use accessible services or technology. A separate item bank for deaf-related experiences was created, reflecting deaf-specific technology such as sharing health-related ASL videos through social network sites and using video remote interpreting services in health settings. After data collection is complete, we will conduct a series of analyses on deaf people’s health information seeking behaviors across technology-mediated platforms.

**Conclusions:**

HINTS-ASL is an accessible health information national trends survey, which includes a culturally appropriate set of items that are relevant to the experiences of deaf people who use ASL. The final HINTS-ASL product will be available for public use upon completion of this study.

## Introduction

The Health Information National Trends Survey (HINTS) is a survey of people’s health information seeking behaviors, including technology mediated sources. HINTS has previously been used for baseline data/endpoints in health communication studies and as outcome measures in several human-computer interaction studies [1]. Instruments are available in English and Spanish [2]. The methodology for translation includes an iterative process of forward and back translation, independent reviews by bilingual experts, and pretesting on a population with characteristics similar to the population to be assessed.

There are currently over 200 published papers using HINTS and over 50,000 participant responses collected. However, HINTS is not available in American Sign Language (ASL) yet, thus excluding deaf adult signers from health communication research. This exclusion has led to a significant gap in knowledge of Internet usage for health information access in this underserved and understudied population. With National Institutes of Health (NIH) funding, we were able to culturally adapt and translate a set of HINTS items to ASL. We also added items that were unique to the deaf signers’ experiences with seeking health information and communicating with health professionals. For example, we included items that asked deaf people about their experiences with using video relay interpreting (VRI) services in medical settings. We also included questions about the modality of communication that they used with their healthcare providers. This is the first nationwide survey that is accessible in ASL and includes items that are culturally relevant to deaf people’s health communication experiences.

This paper describes the cultural adaptation methods used to make existing HINTS items applicable to deaf people. In addition, we describe the linguistic translation methods to translate HINTS items to ASL. We produced an accessible health information seeking survey in ASL (HINTS-ASL) and a culturally appropriate set of items that are relevant to the experiences of deaf adults.

## Methods

To make the HINTS survey accessible in order to gather data for the deaf population in America, we obtained approval from the National Cancer Institute (NCI) HINTS group to translate the items to ASL. Because existing HINTS items were originally written and validated in the general population, we followed the cultural adaptation process to ensure that these items are relevant and can be answered by deaf users of accessible technology and services.

### Addition of New Deaf Health Experience Items and the Cultural Adaptation of Existing HINTS Items

We reviewed HINTS items that required cultural adaptation for deaf people who use accessible technology and services. Some items from the social media section required cultural adaptation for use with deaf people who use accessible services or technology. An example of an original item taken from HINTS and its culturally adapted item is shown below.

Sometimes people use the Internet to connect with other people online through social networks like Facebook or Twitter. This is often called “social media”. In the last 12 months, have you used the Internet to write in an online diary or blog (ie, Web log)?

Original question

In the last 12 months, have you used the Internet to write in a status update on Facebook or to share ASL vlogs?

Adapted question

**Figure 1:**
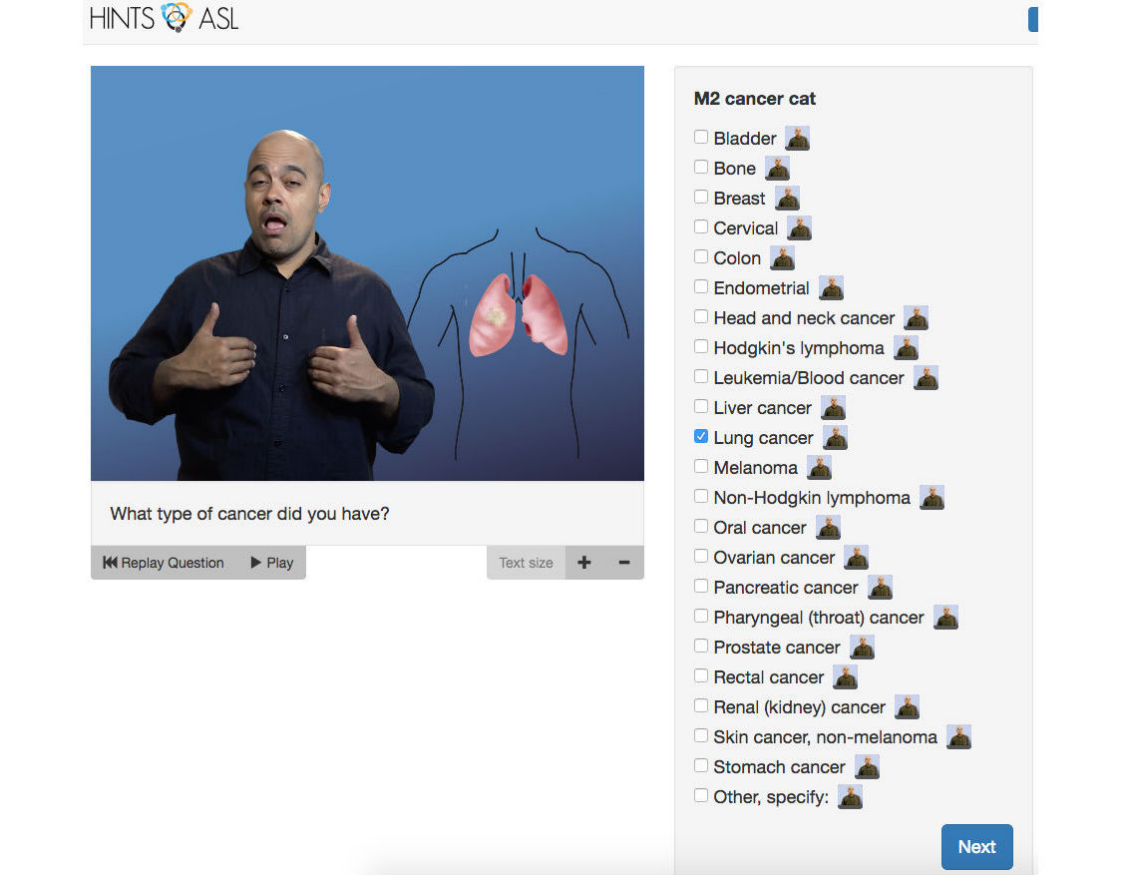
Lung cancer answer option with medical illustration.

**Figure 2:**
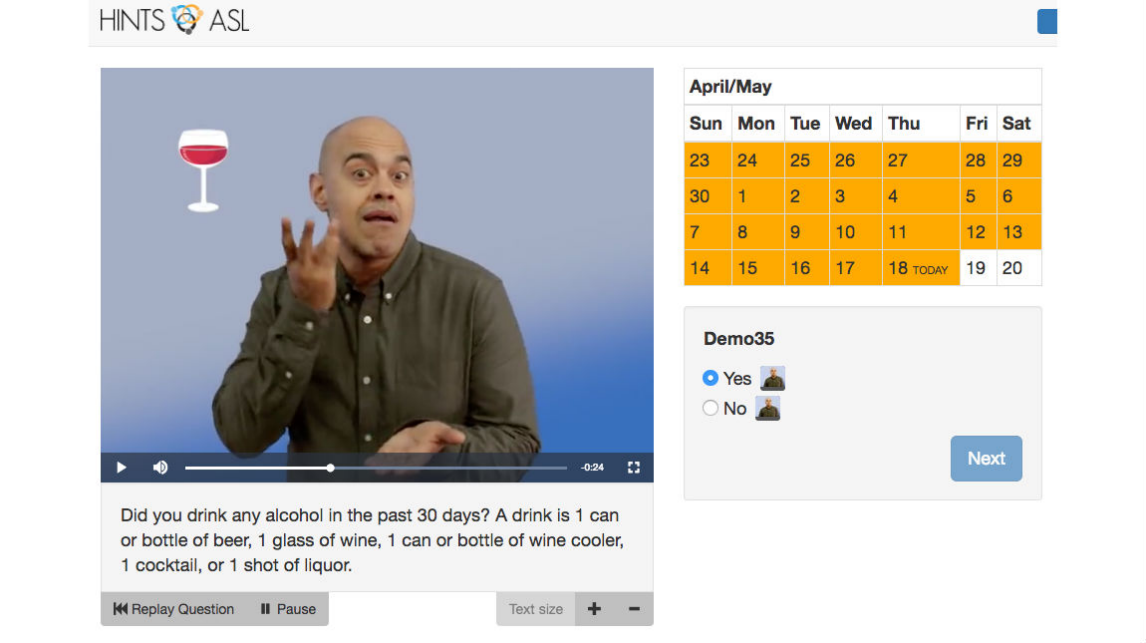
Screenshot of HINTS-ASL item with calendar feature.

Another example of an original HINTS item, “In general, how much would you ‘trust’ information about health or medical topics from each of the following?” required cultural adaptation and translation to be consistent with the TRUST and CONFIDENT sign, which is similar in handshape, location, orientation, and movement [3]. This sign was also used in “Overall, how ‘confident’ are you that you could get advice or information about health or medical topics if you needed it?” The research team met and discussed this issue and came up with the solution of adding a sign of “believe” along with “trust”. We then tested its translation with deaf ASL users who have high school education or lower. All translations were shown online using HINTS-ASL layout (Figures 1 and 2). Each participant played the question and then the response options, all in ASL. No English texts were shown in this testing phase. These participants answered the questions without any difficulty, which validates the linguistic translation of those questions.

New items reflecting deaf-related experiences were added to the existing HINTS item bank, reflecting deaf-specific technology such as VRI services in health settings. A team of experts from national organizations working with deaf people—the National Association of the Deaf, Telecommunications Inc. and the Rehabilitation Engineering Research Center at Gallaudet University—used case studies to draft items related to video remote interpreting and VRI services for health purposes. Each item was evaluated for nomination for inclusion in the survey if it met the following criteria: (1) the item should measure a single target concept, and (2) the item should be relevant to deaf people’s experiences. If the item content was too narrow to have universal applicability to the deaf population that uses ASL, the item was revised or removed. All deaf-related items were tested for cultural relevancy with deaf ASL users.

One of the items that was found to be problematic in ASL translation was “What is your hearing level in your better ear?” The translation of that question was riddled with difficulty, because the phrase “better ear” doesn’t have a direct translation in ASL. Another problem was the typical responses used by audiologists in measuring hearing would be mild, moderate, severe, and profound. The original question in ASL was “Point-to-both-ears, which better, level what?” The problem with the framing of that question, according to the cognitive interviews, was that the respondents would say that they’re not sure and that both ears are deaf, period. They did not know what “level” they were deaf at and that they were simply deaf. One participant said: “I’m 100% deaf in both ears!” and another felt this was an inappropriate question. This led us to think that questions about hearing levels can easily be confused with questions about their cultural identity (eg, deaf, hard-of-hearing, or hearing). This question was then removed from the questionnaire because it was not culturally compatible. In lieu of this audiological-specific question, we used the following more culturally acceptable question:

If a person speaks to you through a combination of listening and/or lip-reading in a quiet room, how much can you understand what the person says?

This included a response set of:

All of what they said.

Most of what they said.

Some to little of what they said.

Did not understand what they said.

Participants in the debriefing process did not indicate any concerns for this question and it was used in lieu of the audiological question to collect information from the participants about their perceived access to auditory information.

The items that are included in the deaf experience item bank are shown in Textbox 1.

**Deaf experience item bank.**

Items

Are any of your immediate family members deaf or hard of hearing?

Which immediate family member is deaf or hard of hearing?

When you were a teenager (between 12 and 18 years old), how well did you understand what your parents said?

How do you communicate with your doctor, nurse, or health professional that you see the most?

Most of the times, the interpreter was… [on site or through VRI]

If you had to choose one, how do you prefer to use an interpreter in health settings?

How would you rate the quality of VRI services you received in healthcare settings in the past 12 months?

Overall, how would you rate the quality of interpreting services you received on site in healthcare settings in the past 12 months?

Overall, how well did you understand your ASL interpreters at your healthcare appointments in the past 12 months?

Overall, how well did the ASL interpreters at your healthcare appointments in the past 12 months understand you?

Have you used video relay services (VRS) to contact your doctor, health insurance, or any medical service?

How often do you struggle or get frustrated when you used VRS to contact your doctor, health insurance or any medical service?

Please think about your most recent frustrating experience with using VRS to contact your doctor, health insurance, or any medical service. What was the main reason for this frustration?

Do you feel having an onsite interpreter in the doctor’s office will interfere with your disclosure of health information with the doctor?

Do you feel having a VRI will interfere with your disclosure of health information with the doctor?

If a person speaks to you through a combination of listening and/or lip-reading in a quiet room, how much can you understand what the person says?

Which language do you prefer or feel comfortable using?

What do you identify yourself as? Culturally Deaf, deaf, hard of hearing, or hearing?

### Linguistic Validation and Translation

The backward and forward translators involved in this project were deaf native signers, born to parents who were deaf or had older deaf siblings and thus were exposed to ASL since birth. They were also proficient in English. The translation team consisted of 2 forward translation consultants (A and B) and a backward translation consultant. All were bilingual in ASL and English with experience translating test items. The concept of linguistic validation was explained to the team in detail before working on the translations. Forward translation consultant A was given a list of HINTS items in English to be translated to ASL. A video camera was used to record the translation. A backward translator viewed forward translation A’s videos and wrote down the equivalent items in English. A reconciliation of items was then performed by the principal investigator. Items that failed the reconciliation process were then passed over to forward translation consultant B who translated these items to ASL using a video camera. The backward translator viewed translation B’s ASL videos and made further corrections to the back-translated English items. A deaf oncology nurse who is bilingual in ASL and English reviewed the translations to ensure concept equivalence of cancer-related items were delivered in ASL. A native ASL user with background training in ASL linguistics assumed the model signer role for the final HINTS-ASL version.

The final HINTS-ASL items included grammatically correct ASL, for example, including a question marker at the end indicated by raising the eyebrows. These subtle changes in facial expressions convey important linguistic information in ASL and the equivalence in spoken English would include the raising of pitch at the end of a sentence to indicate a question. Other linguistic and cultural modifications to the translations included the questions regarding the timeline, such as in the last 12 months. The research team felt that “last 12 months” in English should be converted to “since 1 year” in ASL, due to the concern that the participants may misinterpret “last 12 months” as January of the past year, even if the year has not fully ended. Those new phrases in ASL were tested during the cognitive debriefing sessions and responses supported the alternate word choices. All timeline questions were then modified to the culturally and linguistically appropriate phrases of “since 1 year” in ASL.

### Cognitive Debriefing

The goals of the cognitive debriefing interviews were to assess whether (1) the respondents understood the intent of the HINTS-ASL questions; and (2) the questions were both culturally acceptable and contextually relevant to deaf signers’ experiences. The English version of the survey items on ASL video clips were not shown to the participants to ensure focus on the clarity of ASL items. If the participant had any difficulty with understanding the items, the interviewer provided alternative words or concepts, or asked the person to propose improvements. By having the test items audited and validated by members of the target language community—the deaf community—they align with Harris and colleagues’ terms of ethics on how a researcher can ethically work within the deaf community, a marginalized group [4].

As an effort to save cost in re-filming and re-editing ASL video clips, an open-ended cognitive interview approach with the HINTS-ASL items was utilized, allowing time for follow-up, clarification, and expansion on the test items with the respondents if and when needed. A native speaker of ASL and a Certified Deaf Interpreter (CDI) with a doctoral degree in educational linguistics, specializing in ASL discourse with an extensive background in ASL pedagogy, translation, and interpretation led this process. As a CDI, she has significant experience working with a variety of clients ranging from those who are semi-lingual to multilingual in multiple sign languages in medical, mental, and health-related settings.

Following approval from human participants review board and after written consent was obtained from participants, 1- to 2-hour cognitive debriefing interviews were conducted with a target linguistic community of deaf people who use ASL primarily and have a high school degree or less. Involving members of the deaf community with high school education or less in this cognitive debriefing process also helps to ensure the test items are understood by the greater majority of the deaf community, increasing the reliability and validity of the test items across a larger number of participants. Cognitive debriefing was performed through 3 waves of face-to-face interview sessions with 4 to 5 ASL signers per wave. Each wave included both male and female signers with a high school degree or lower.

### Web-Based App Development and Testing

AllOut Marketing, Inc. was contracted to iteratively develop the Web-based app and the user interface testing was done at the authors’ institution. Since the videos were signed in ASL, the human-computer interaction played a significant role in having participants not only understand what the question is asking, but also feeling comfortable with the visual layout on the screen. In the user experience sessions, participants often asked the research staff to clarify what or where a “kidney” was, or what a “regular dial-up telephone line” looked like. When the researcher asked how it could be improved, they recommended visual aid to be placed adjacent to the signer. We incorporated their feedback to improve the video (Figure 1). The pictures helped greatly with the clarity and understanding of the questions in the next wave of user experience sessions.

A person who listens or reads French may respond to questions similarly to a person who listens or reads English. A deaf person who uses ASL should not be viewed differently from those readers as far as giving certain information. The only difference is that the deaf person sees the ASL videos on the computer and selects responses on the screen. The hearing person sees the English/French words on a paper and marks responses. Sign languages are not permanently embedded on the screen and are ephemeral, requiring the participant to press a button to play the question (or option) again to review information that was presented. In other words, after you watch a person narrate a question or a sentence, the information disappears. Languages that have a written version can be permanently left on the screen and easily revisited as one reads the options and then looks back at the question if needed. This type of question format was not as viable in ASL, due to its ephemeral nature, and needed to be repeated for each option. For instance:

In general, how much would you trust information about cancer from a doctor?

In general, how much would you trust information about cancer from family or friends?

Therefore, the online survey app was designed to allow replays of the question and response options.

An item example, which offers an option of watching the ASL version of the item on a video or turning the video off for greater emphasis on the English text, is shown in Figure 2. A highlighted calendar next to the video and English text provided added visual information on the range of days that the experience or symptom may have or may not have occurred. Participants could also pause the video to take a closer or longer look at the image before proceeding to answer. The final HINTS-ASL was pre-tested with 4 deaf adults to determine whether the survey is of a suitable length to avoid participant burden. Then the Web-based app was finalized and prepared for administration in future studies.

### Administration

All research staff who participated in administering surveys were required to complete human participants training and were trained in conducting informed consents. The interviewers were also trained to know each question asked in the survey and how to correctly translate them into ASL without losing the meaning of the original English question. When prospective participants expressed interests in taking the survey, they were asked to watch informed consent in ASL and provide written consent if they wished to participate. The researcher then met with the participant to review their rights. Participants were assured that all information provided by them will be kept confidential and access to their data was controlled by the principal investigator. The participants were also informed that the interviewers did not have access to the dataset and could not see the participants’ responses. Deaf participants were also told that, for dissemination purposes, results were presented in aggregate so that no person is identifiable.

A purposive sampling method was used to ensure similar distribution of key demographic characteristics such as US region, age, and education. The US census was used to determine estimates for each region, age group, and education level. We gathered information on deaf-specific demographics as well. Through channels targeting the Deaf community, we solicited participation using methods that we have used in the past with success, including distributing flyers, through word of mouth in the community, community centers and churches, deaf organizations, creating a Facebook page for the study, and in local or statewide deaf-oriented listserves. Communication with community recruiters and interested participants also took place through community events, email, Facebook, and Facetime/videophone.

Data was collected in ASL by one of the following methods to meet the needs of the diverse deaf sample: (1) interviewer-guided survey in person; or (2) interviewer-guided survey by videophone. For the first method, interview-guided survey in person, the respondent may have opted to view the questions in ASL and enter responses directly while the interviewer stands by to aid if required or to have the interviewer ask the questions and then enter the participant’s responses. For the second method, the interview-guided survey by videophone, the interviewer checked in with the participant to ensure they understood the informed consent that was shown through ASL videos online and briefly reviewed their rights that were explained in the informed consent. When the participant was ready to begin the survey, the interviewer sent the survey link via email. The interviewer remained visible on videophone to answer questions during the session, including immediate assistance of technical difficulties.

**Figure 3:**
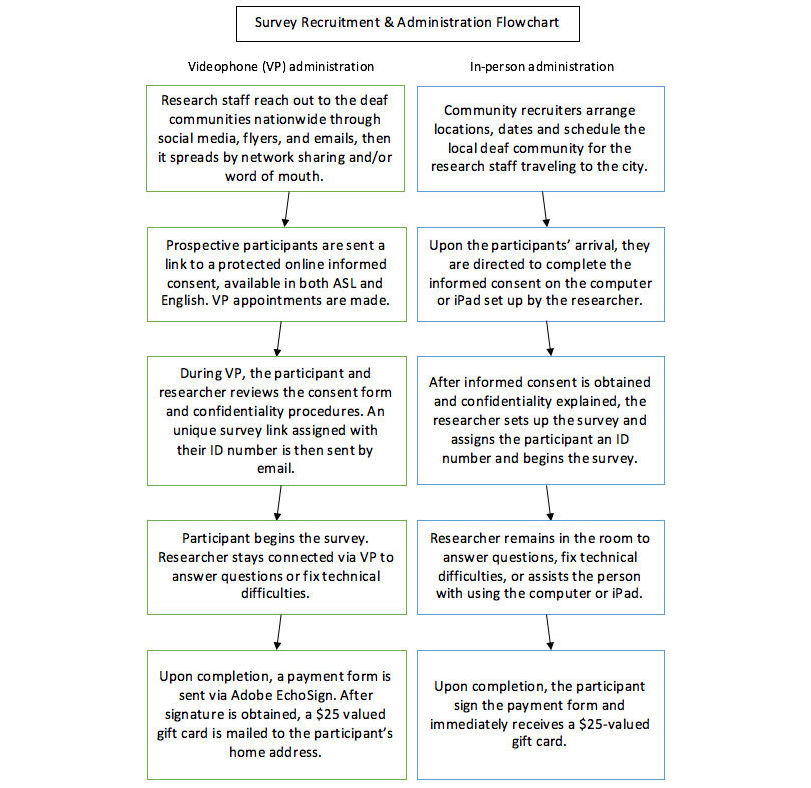
Flowchart for survey administration and recruitment.

On average, it took approximately 45 to 60 minutes to complete the online survey. Upon completion, each participant was compensated a US $25 gift card as a gratuity for their time and participation. The flowchart procedure for each method is shown in Figure 3.

## Results

Data collection is ongoing. At the time of this publication, a total of 1356 deaf adults provided informed consent and took the survey. An unweighted summary of the demographic data is shown in Table 1. A large number came from the South (39.90%, 541/1356), followed by West (26.18%, 355/1356), Northeast (10.77%, 146/1356), and Midwestern (23.16%, 314/1356). Within the sample ranging from 18 to 95 years old that had a mean age of 50 (SD 18) years, the 18 to 34 age group had the largest number of participants (32.60%, 442/1356) followed by the 35 to 49 age group (25.22%, 342/1356). Half of the HINTS-ASL sample had a college degree. This sample included 16.15% (219/1356) who self-identified as lesbian, gay, or bisexual and 36.43% (494/1356) who were people of color. When asked about the hearing status of the respondent’s parents, 23.38% (317/1356) reported having parents who are deaf.

Approximately 38.79% (526/1356) earned less than US $35,000. Nearly 87.09% (1181/1356) of the deaf sample reported having health insurance coverage and 62.54% (848/1356) had a healthcare provider that they see regularly. Although most of the sample (66.96%, 908/1356) reported having been told by their healthcare provider to have a medical diagnosis (ie, hypertension, diabetes, depression/anxiety disorder, etc), and many rated their health as “good” (34.59%, 469/1356) or “very good” (37.54%, 509/1356).

**Table 1:** Sociodemographic characteristics (N=1356).

Characteristic		n (%^a^)
Age in years, mean (SD)		50 (18)
**Gender**		
	Male	567 (41.81%)
	Female	776 (57.23%)
	Missing/did not answer	13 (0.96%)
**Age group, years**		
	18-34	442 (32.60%)
	35-49	343 (25.29%)
	50-64	325 (23.97 %)
	65-74	172 (12.68%)
	75 and over	74 (5.46%)
**Ethnicity/race**		
	Hispanic	189 (13.94%)
	Non-Hispanic White	845 (62.32%)
	Non-Hispanic Black or African American	156 (11.50%)
	Other	149 (10.99%)
	Missing/did not answer	17 (1.25%)
**Geographic location**		
	Northeast	146 (10.77%)
	South	541 (39.90%)
	Midwestern	314 (23.16%)
	West	355 (26.18%)
**Education**		
	Less than high school	54 (3.98%)
	High school graduate	331 (24.41%)
	Some college	301 (22.20%)
	College graduate	667 (49.19%)
	Missing/did not answer	3 (0.22%)
**Preferred language**		
	ASL^b^	716 (52.80%)
	Both ASL and English	624 (46.02%)
	Missing/did not answer	16 (1.18%)
**Occupation**		
	Employed	663 (48.89%)
	Unemployed	126 (9.29%)
	Homemaker	62 (4.57%)
	Student	164 (12.17%)
	Retired	259 (19.10%)
	Disabled	37 (2.73%)
	Other	11 (0.81%)
	Missing/did not answer	34 (2.51%)
**Personal history of cancer**		
	Yes	175 (12.91%)
	No	1129 (83.26%)
	Missing/did not answer	51 (3.76%)

^a^Unweighted percent.

^b^ASL: American Sign Language.

## Discussion

### Transformative Research Paradigm

The additional step of conducting cognitive interviews prior to translations was rooted in the transformative paradigm [5], where researchers incorporate elements of social justice and human rights in the research process by involving the target community in the validation of the survey items. In the transformative research paradigm, researchers make explicit the issue of power and who holds the power, but also explicitly identify the process of sharing ownership of the research process with the research team, participants, and the target community [6,7]. In any research involving the deaf community, deaf people must be involved in every step of the research, and not simply as research assistants [4]. In the case of the HINTS-ASL research project, almost all members of the research team were deaf and led by a primary investigator who is deaf and bilingual in ASL and English. The hierarchical structure typically associated with research was destabilized in this project, and made more equal in this process where the deaf primary investigator, being a person of color, worked closely with a deaf team of experts, with at least half of the research team members being deaf people of color. These experts worked closely with members of the deaf community to ensure that the survey items in ASL were still accessible to and understood by deaf signers who have high school education or lower. These deaf community members who participated in evaluating the ASL items have high school degree or lower. All investigations in indigenous communities, such as the deaf community, should be done with the deaf community’s consent and with the deaf community’s joint control and guidance [8], conditions of which were met in this study.

The involvement of participants in this process is transformative for both the research team and the participants because it redistributes the power back to the community by having the participants provide input on the test items. Regardless of the results, the research team, participants, and the community were in some ways transformed by the research process, hence the name of the transformative research paradigm [5].

### Conclusion

HINTS-ASL is an accessible health information national trends survey, which includes a culturally appropriate set of items that are relevant to the experiences of deaf people who use ASL. The final HINTS-ASL product will be available for public use upon completion of this study.

## Abbreviations

ASL: American Sign Language
CDI: Certified Deaf Interpreter
HINTS: Health Information National Trends Survey
HINTS-ASL: Health Information National Trends Survey in American Sign Language
NIDCD: National Institute on Deafness and Other Communication Disorders
NIH: National Institutes of Health
VRI: video relay interpreting
VRS: video relay services

## Appendix

Multimedia Appendix 1 Peer review report.
