# Supplementary material for: Health Information National Trends Survey in American Sign Language (HINTS-ASL): Protocol for the Cultural Adaptation and Linguistic Validation of a National Survey
Source: JMIR Res Protoc. 2017 Sep 13;6(9):e172. doi: 10.2196/resprot.8067 (PMC5617902; doi:10.2196/resprot.8067)
Supplement: Multimedia Appendix 1 [file resprot_v6i9e172_app1.pdf]

RESUME AND SUMMARY OF DISCUSSION: This application requests support to adapt the Health Information National Trends Survey (HINTS) for American Sign Language users. If successful, the findings will identify barriers to obtaining health-related information on the internet experienced by people who are deaf or hard of hearing (D/HH) and provide insights into building communication models to better reach this population. The study has a high potential impact. In discussion, reviewers identified application strengths that include a promising young investigator, a significant focus and a rigorous approach that will provide valuable information for the deaf community and their health care providers. The project does an excellent job of including undergraduate students in the research, who themselves are deaf/HOH and enhancing the research environment at this institution. Reviewers also identified some minor weaknesses. These include the need to provide a better rationale for targeting cancer, address concerns regarding data collection and methods and other minor weaknesses noted below. Overall, this is an outstanding application with strengths that include the team and research environment and plan to include D/HH students that far outweigh the noted correctable weakness.

DESCRIPTION (provided by applicant): NCI's Health Information National Trends Survey is heavily dependent on English, which present serious language barriers to Deaf patients who use American Sign Language (ASL). The availability of HINTS in ASL and English and such that is valid for users of accessible technology and services will provide important insights on understanding the trends in Deaf people's use of the Internet for health-related purposes and improving health communication models that will lead to better personal and public health within the underserved deaf population. The research plan builds on Dr. Kushalnagar's prior research on health communication and quality of life outcomes with Deaf population. The proposed research plan involves a development component (translation) and a research component (health information). Specific aims include (1) translating and implementing HINTS survey in ASL and (2) describing the trends in Deaf sub-groups' use of the Internet for cancer health information. The study will be conducted by a research team that includes: an early career investigator with expertise in health communication and quality of life outcomes in DHH population, a senior investigator with expertise in cancer control and Deaf community outreach, a biostatistician, and a team of Deaf/HH undergraduate student researchers. Results from this study will provide a better understanding of the trends in using the Internet for health-related purposes, which may vary across Deaf sub-groups. The goals of this application are consistent with the NIDCD 2012-2016 strategies related to improving outcomes for human communication and reducing health disparities, and expected findings will support improvement and refinement of health websites that utilize ASL cancer health videos for Deaf consumers, with goals of maximizing effectiveness of dissemination for health-related purposes.
